# Unravelling myalgic encephalomyelitis/chronic fatigue syndrome (ME/CFS): Gender‐specific changes in the microRNA expression profiling in ME/CFS

**DOI:** 10.1111/jcmm.15260

**Published:** 2020-04-14

**Authors:** Amanpreet K. Cheema, Leonor Sarria, Mina Bekheit, Fanny Collado, Eloy Almenar‐Pérez, Eva Martín‐Martínez, Jose Alegre, Jesus Castro‐Marrero, Mary A. Fletcher, Nancy G. Klimas, Elisa Oltra, Lubov Nathanson

**Affiliations:** ^1^ Institute for Neuro Immune Medicine Dr. Kiran C. Patel College of Osteopathic Medicine Nova Southeastern University Fort Lauderdale FL USA; ^2^ Department of Nutrition Dr. Kiran C. Patel College of Osteopathic Medicine Nova Southeastern University Fort Lauderdale FL USA; ^3^ Halmos College of Natural Sciences and Oceanography Nova Southeastern University Fort Lauderdale FL USA; ^4^ Department of Veterans Affairs Miami VA Healthcare System, Research Service Miami FL USA; ^5^ South Florida Veterans Affairs Foundation for Research and Education Inc Fort Lauderdale FL USA; ^6^ Escuela de Doctorado Universidad Católica de Valencia San Vicente Mártir Valencia Spain; ^7^ National Health Service Manises Hospital Valencia Spain; ^8^ Vall d'Hebron University Hospital Vall d'Hebron Research Institute Universitat Autónoma de Barcelona Barcelona Spain; ^9^ School of Medicine Universidad Católica de Valencia San Vicente Mártir Valencia Spain

**Keywords:** exercise, gender, immune function, inflammation, microRNAs, myalgic encephalomyelitis/chronic fatigue syndrome, peripheral blood mononuclear cell

## Abstract

Myalgic encephalomyelitis/chronic fatigue syndrome (ME/CFS) is a multisystem illness characterized by medically unexplained debilitating fatigue with suggested altered immunological state. Our study aimed to explore peripheral blood mononuclear cells (PBMCs) for microRNAs (miRNAs) expression in ME/CFS subjects under an exercise challenge. The findings highlight the immune response and inflammation links to differential miRNA expression in ME/CFS. The present study is particularly important in being the first to uncover the differences that exist in miRNA expression patterns in males and females with ME/CFS in response to exercise. This provides new evidence for the understanding of differential miRNA expression patterns and post‐exertional malaise in ME/CFS. We also report miRNA expression pattern differences associating with the nutritional status in individuals with ME/CFS, highlighting the effect of subjects' metabolic state on molecular changes to be considered in clinical research within the NINDS/CDC ME/CFS Common Data Elements. The identification of gender‐based miRNAs importantly provides new insights into gender‐specific ME/CFS susceptibility and demands exploration of sex‐suited ME/CFS therapeutics.

## 1 INTRODUCTION

The underlying mechanisms associated with the onset and progression of myalgic encephalomyelitis/chronic fatigue syndrome (ME/CFS), a condition characterized by an abrupt or delayed onset of persistent or relapsing symptomatology, including memory problems, muscle and joint pain, gastrointestinal issues, neurological problems, hormonal imbalance and debilitating fatigue or easy fatigability, remains unclear. Still, a decade of research into ME/CFS disease activity has provided evidence of immune dysfunction described by persistent immune activation associated with the onset and progression of the illness.^1^, ^2^, ^3^, ^4^, ^5^, ^6^, ^7^, ^8^, ^9^, ^10^

During the last decade, miRNAs have been emerging as biomarkers in ME/CFS due to their regulatory role in the development, maturation and proliferation of immune cells.^11^, ^12^, ^13^, ^14^, ^15^, ^16^, ^17^ Brenu et al reported miR‐127‐3p, miR‐142‐5p and miR‐143‐3p as potential plasma and miR‐146a, miR‐223 and miR‐21 as cytotoxic lymphocyte biomarkers for ME/CFS diagnosis,^15^, ^16^ and due to this illnesses’ similarity with fibromyalgia, a complex neuroimmune condition characterized by similar symptomatology, miR‐223‐3p, miR‐451a, miR‐338‐3p, miR‐143‐3p and miR‐145‐5p have come into focus as well.^17^, ^18^ Petty et al^19^ reported up‐regulated miR‐99b, miR‐330, miR‐126 and miR‐30c as potential diagnostic markers for individuals with ME/CFS among the 34 miRNA which were found to be differentially expressed in individuals with ME/CFS. A recent study reported significantly diminished miR‐let‐7i‐5p, miR‐93‐3p and miR‐200a‐5p in cerebrospinal fluid of ME/CFS subjects who underwent exercise challenge^20^ which is important due to the post‐exertional malaise (PEM) being a unique characteristic of ME/CFS. Altogether, despite of the evidence of gender‐related differences in this illness,^21^, ^22^ a comprehensive analysis of the effects of an exercise testing on differential miRNA expression between males and females with ME/CFS has not been performed so far.

The present study aimed to investigate peripheral blood mononuclear cells (PBMCs) miRNA expression in ME/CFS subjects in comparison with sedentary healthy controls (HCs) under an exercise challenge. The differential expression of miRNAs observed in ME/CFS was further evaluated in females and males. Our results indicate that miRNA expression differs between female and males with ME/CFS both at the baseline, and in response to the exercise challenge. In addition, we found that miRNA expression was different in PBMCs of fasting as compared to non‐fasting subjects with ME/CFS, which emphasizes the importance of the National Institute of Health (NIH), National Institute of Neurological Disorders and Stroke (NINDS) and Centers for Disease Control and Prevention (CDC)’s newly adopted ME/CFS Common Data Elements (CDE) principles for detailed reporting of subjects’ conditions.

## 2 METHODS

### 2.1 Participants and samples

A community‐based cross‐sectional study included 63 clinically diagnosed ME/CFS subjects and 55 healthy controls matched for age (±5 years) and BMI (±2). All individuals with ME/CFS and HCs were recruited from three different geographical areas: Miami/Fort‐Lauderdale, Florida, USA, and Barcelona and Valencia, Spain, as part of larger biomarker‐oriented studies. For inclusion, all ME/CFS subjects met the 1994 CDC/Fukuda and 2003 Canadian Case definitions for ME/CFS.^6^, ^23^, ^24^, ^25^ Major exclusion criteria included: active smoking or alcohol history, diabetes, immunodeficiency disorders, cardiovascular diseases, stroke, autoimmune conditions, malignancy or systemic infection for at least 2 weeks before blood sample collection. All female subjects were asked to complete the Gynecologic Questionnaire to assess routine gynecologic parameters and were asked to come for the assessment and collection of blood during the first two weeks of their menstrual cycle, if pre‐menopausal. The details on health‐related quality of life and exercise challenge are provided in Appendix S1 under measures.

### 2.2 Isolation of PBMCs and RNA extraction

Up to 8 mL of whole blood/sample were collected in K_2_EDTA tubes (Becton Dickinson) and processed within 2 hours by dilution at 1:1 (v/v) ratio in phosphate‐buffered saline solution (PBS) with layering on top of 1 volume of Ficoll‐Paque Premium (GE Healthcare) and separation by density centrifugation at 500× *g* for 30 minutes (20°C, brakes off). PBMC layers were isolated and washed with PBS. Isolated PBMC pellets were resuspended in 1 volume of red blood cell lysis buffer (155 mmol/L NH_4_Cl, 10 mmol/L NaHCO_3_, 0.1 mmol/L EDTA and pH 7.4), kept on ice for 5 minutes, and centrifuged (20°C at 500× *g* for 10 minutes). Washed pellets were re‐suspended in freezing medium (90% FBS, 10% DMSO) adjusting their concentration to 10^7^ cells/ml, aliquoted and frozen in liquid nitrogen until use. Total RNA was extracted using RNAzol (Molecular Research Center, Inc.) according to the manufacturer's instructions. RNA quality was assessed using Agilent TapeStation 4200 (Agilent). All RNA samples had RNA Integrity number (RIN) above 7.

### 2.3 miRNA expression analysis

We used NanoString nCounter platform (NanoString Technologies) to evaluate miRNA expression with the nCounter Human miRNA Panel v3 (NanoString Technologies, GXA‐MIR3‐12). The panel included unique oligonucleotide tags for over 800 highly curated human miRNAs (from miRbase v21) and five housekeeping genes for normalization of expression between samples (ACTB, B2M, GAPDH, RPL19 and RPLP0). Each sample was analysed by using 100 ng of total RNA for processing and consecutive hybridization (21 hours at 65°C) to the probe pairs consisting of Reporter Probe, which carry the signal on its 5′ end, and Capture Probe, which holds biotin on its 3′ end. After hybridization, sample cleanup and counting were performed according to the manufacturer's instructions.

### 2.4 Data analysis

Raw counts were analysed using NanoString nSolver version 4.0 software. We calculated geometric means of negative ligation controls plus two standard deviations for all samples. All count values below this threshold were excluded from normalization. After that, all the normalization steps were performed according to manufacturers' instructions.

Only miRNAs that were expressed in 75% or more of samples in at least one group were further analysed. We then selected miRNAs that showed statistical significance with *P *<* *.05. Differentially expressed miRNAs in each contrasted group were detected by one‐way ANOVA using Partek Genomic Suite v7, and false discovery rate (FDR) was calculated for multiple comparisons using the *q*‐value.^26^ MiRNAs were differentially expressed if they met the following selection criteria: FDR ≤ 0.1, and fold change (FC) of at least 1.5 in either direction. For some further analyses, we also considered miRNAs that did not pass FDR ≤ 0.1 but passed unadjusted *P *<* *.05 (as indicated in Tables S1‐S5 and explained in the Section 3). Data were plotted using GraphPad Prism 5 (GraphPad Software). Age, BMI and SF‐36 values of the participants were examined with Student's *t* test for continuous and chi‐squared test for categorical variables using IBM Statistical Package for the Social Science version 25 (SPSS Inc). The significance level for all analyses was set at *P *<* *.05.

## 3 RESULTS

### 3.1 Participant characteristics

The study compared 37 female and 26 male ME/CFS subjects with 33 female and 22 male‐matched HCs. A total of 24 female and 11 male ME/CFS subjects, and 21 female and 13 male‐matched HCs underwent exercise challenge (Table 1a). Blood was drawn from these participants for PBMC isolation at T0 (baseline before the exercise), T1 (VO_2_ max, peak of exercise challenge) and T2 (four hours after T1, recovery). These individuals had unified breakfast (one banana and a yogurt) 30 minutes before the exercise challenges and the first blood draw. T0 was designated for participants at baseline that did not undergo exercise challenge and fasted overnight before blood draw. In the fasting group, there were 13 female and 15 male ME/CFS subjects with 12 females and 9 males as HCs (Table 1b). No significant difference was observed for age, gender and body mass index (BMI) between individuals with ME/CFS and HCs, regardless of fasting status. ME/CFS cohort had poor (self‐reported, *P* < .05) outcomes in all reported domains of SF‐36 except mental health (*P* = .595), as compared to healthy controls, regardless of fasting status (Table 1).

**TABLE 1:** Baseline clinical characteristics of the study sample (a) who had breakfast; (b) who fasted

(a)			
Variables	ME/CFS (n = 35)	HC (n = 34)	*P* value
Age (y)	46.60 ± 10.52	47.06 ± 11.39	.837
Gender, Female, n (%)	24 (68.57)	21 (61.76)	.667
BMI (kg/m^2^)	26.91 ± 5.63	26.58 ± 4.49	.606
Physical health			
Physical functioning	43.46 ± 36.74	88.63 ± 23.46	**.001**
Role physical	26.07 ± 33.06	80.62 ± 33.04	**.005**
Bodily pain	50.36 ± 28.87	82.14 ± 24.28	**.005**
General health	33.06 ± 19.55	71.69 ± 23.46	**.002**
Mental health			
Vitality	33.28 ± 24.49	70.30 ± 21.17	**.007**
Social functioning	39.61 ± 24.5	81.82 ± 24.62	**<.001**
Role emotional	53.33 ± 39.92	88.54 ± 24.83	**.023**
Mental health	55.55 ± 30.38	71.32 ± 27.61	.595

(b)			
Variables	ME/CFS (n = 28)	HC (n = 21)	*P* value
Age (y)	54.30 ± 7.97	48.72 ± 5.43	.510
Gender, Female n (%)	13 (53.6)	12 (57.14)	.458
BMI (kg/m^2^)	25.23 ± 3.45	24.85 ± 3.17	.433
Physical health			
Physical functioning	26.68 ± 22.52	96.33 ± 3.12	**.003**
Role physical	8.96 ± 27.38	96.57 ± 7.39	**<.001**
Bodily pain	16.04 ± 17.92	90.14 ± 12.66	**.008**
General health	19.68 ± 12.97	79.67 ± 9.18	**.008**
Mental health			
Vitality	15.14 ± 16.52	79.05 ± 9.45	**.003**
Social role function	25.71 ± 25.42	97.41 ± 6.31	**.002**
Emotional function	29.12 ± 27.66	93.55 ± 22.63	**<.001**
Mental health	39.96 ± 26.83	83.81 ± 8.57	.333

Note

Data are shown as mean ± standard deviation (SD) for continuous values and as numbers of cases (percentages) for categorical values. ME/CFS cases vs healthy control subjects. Significant *P*‐values < 0.05 are shown in bold.

Abbreviations: BMI, body mass index; HC, healthy controls; ME/CFS, myalgic encephalomyelitis/chronic fatigue/syndrome; SF‐36, 36‐item Short Form Health Survey.

### 3.2 MicroRNA expression profile differences between ME/CFS individuals and HCs without stratification

**FIGURE 1:**
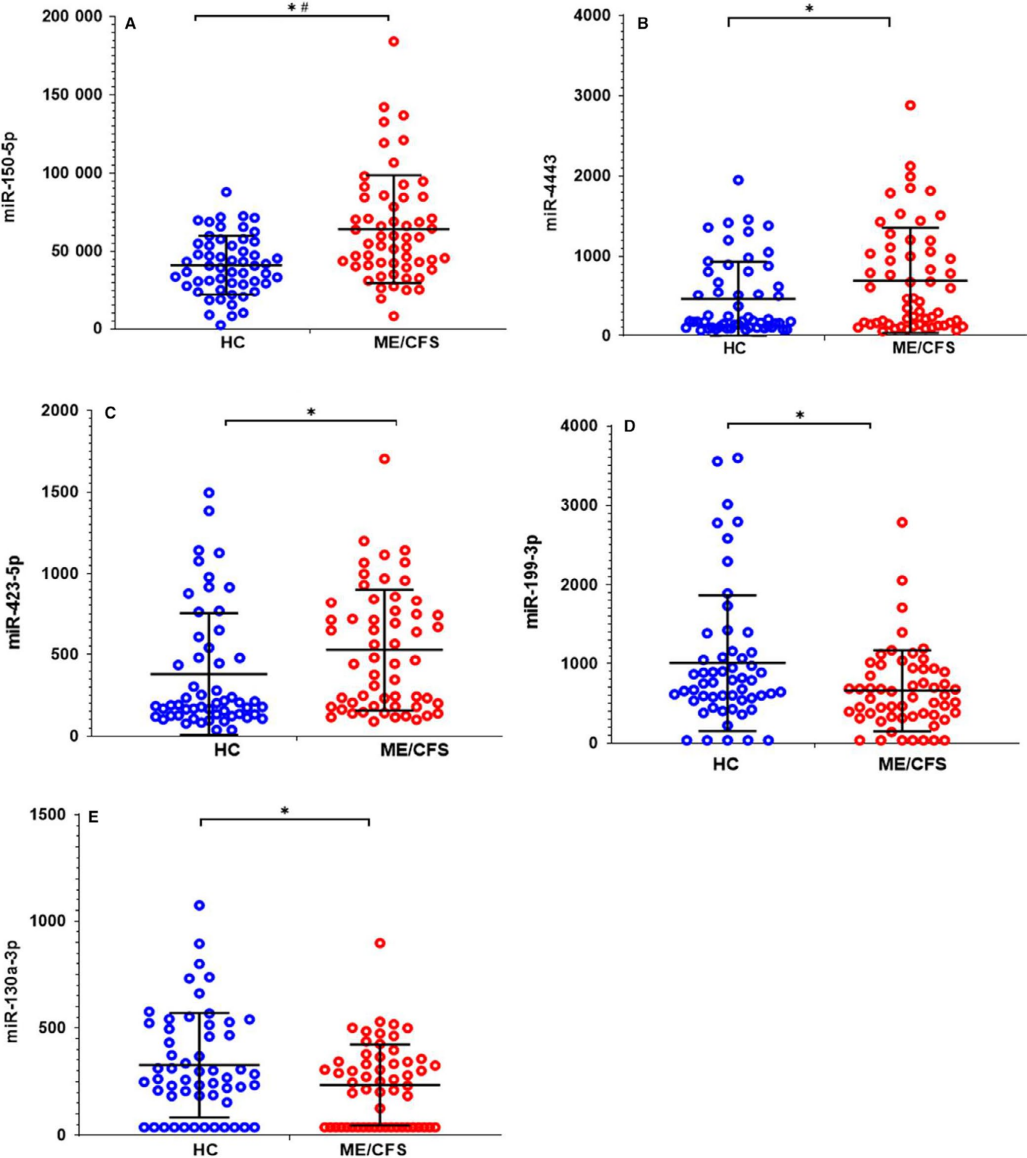
miRNA expression profile differences between diagnosed ME/CFS individuals and HCs without stratification by gender or fasting status. Data are shown as normalized counts for ME/CFS (red) and healthy controls (HC; Blue) at T0 for (A) miR‐150‐5p, (B) miR‐4443, (C) miR‐423‐5p, (D) miR‐199‐3p and (E) miR‐130a‐3p, regardless of fasting status. * denotes *P *< .05 and # denotes FDR < 0.1

We analysed the difference in miRNA expression between all individuals with ME/CFS and HCs regardless of gender and fasting status (breakfast vs fasting). At T0, the expression of miR‐150‐5p, miR‐4443, miR‐423‐5p and miR‐342‐3p was higher, whereas, for miR‐199‐3p, miR‐126a‐3p, let‐7i‐5p and miR‐130a‐3p, the expression was lower in ME/CFS than HCs. However, out of a total of eight miRNAs that were differentially expressed with *P *< .05, only miR‐150‐5p passed criteria of FDR < 0.1 (Figure 1, Table S1a).

Only miRNAs that showed *P* < .05 in non‐stratified and at least once in gender‐stratified analysis were shown in figures.

### 3.3 MicroRNA expression stratified by gender

**FIGURE 2:**
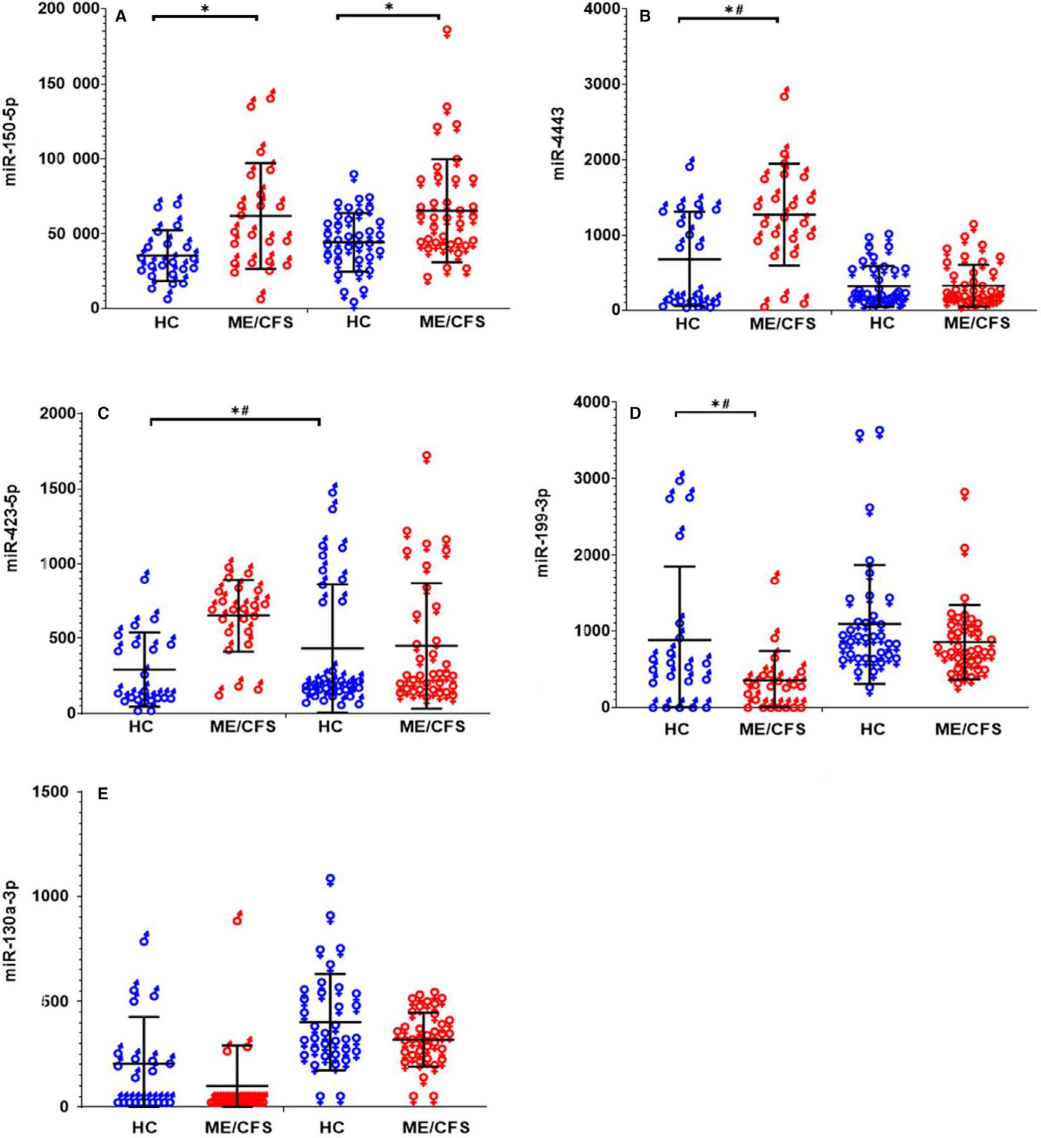
miRNA expression profile differences between diagnosed ME/CFS individuals and HCs at T0. Data are shown as normalized counts for males (♂) and females (♀) ME/CFS (red) and healthy controls (HC; Blue) at T0 for (A) miR‐150‐5p, (B) miR‐4443, (C) miR‐423‐5p, (D) miR‐199‐3p and (E) miR‐130a‐3p, regardless of fasting status. * denotes *P* < .05 and # denotes FDR < 0.1

Only two miRNAs (miR‐150‐5p and miR‐342‐3p) were differentially expressed between ME/CFS females and healthy females (*P* < .05), but none passed criteria FDR < 0.1 (Figure 2, Table S1b). On the other hand, a total of 11 miRNAs were differentially expressed between males with ME/CFS and HCs (*P* < .05) regardless of fasting status and eight passed criteria of FDR < 0.1 (Table S1c). The expression of miR‐423‐5p, miR‐296‐5p and miR‐4443 was higher, whereas miR‐223‐3p, miR‐199‐3p, miR‐16‐5p, miR‐142‐3p and let‐7g‐5p expression was lower in ME/CFS males than HC males. Although miR‐150‐5p did not reach FDR < 0.1, its expression was higher in ME/CFS males than healthy males (*P* < .05, Figure 2).

### 3.4 Response to exercise without stratification by gender

For these comparisons, we only selected data from individuals who completed all three time‐points in exercise and did not undergo extended fasting before blood draw. For figures and discussion, we focused on miRNAs highlighted across analyses showing *P* < .05 in non‐stratified analysis and at least once in sex‐stratified analysis (*P* < .05).

#### 3.4.1 Comparisons for miRNAs expression in response to exercise

**FIGURE 3:**
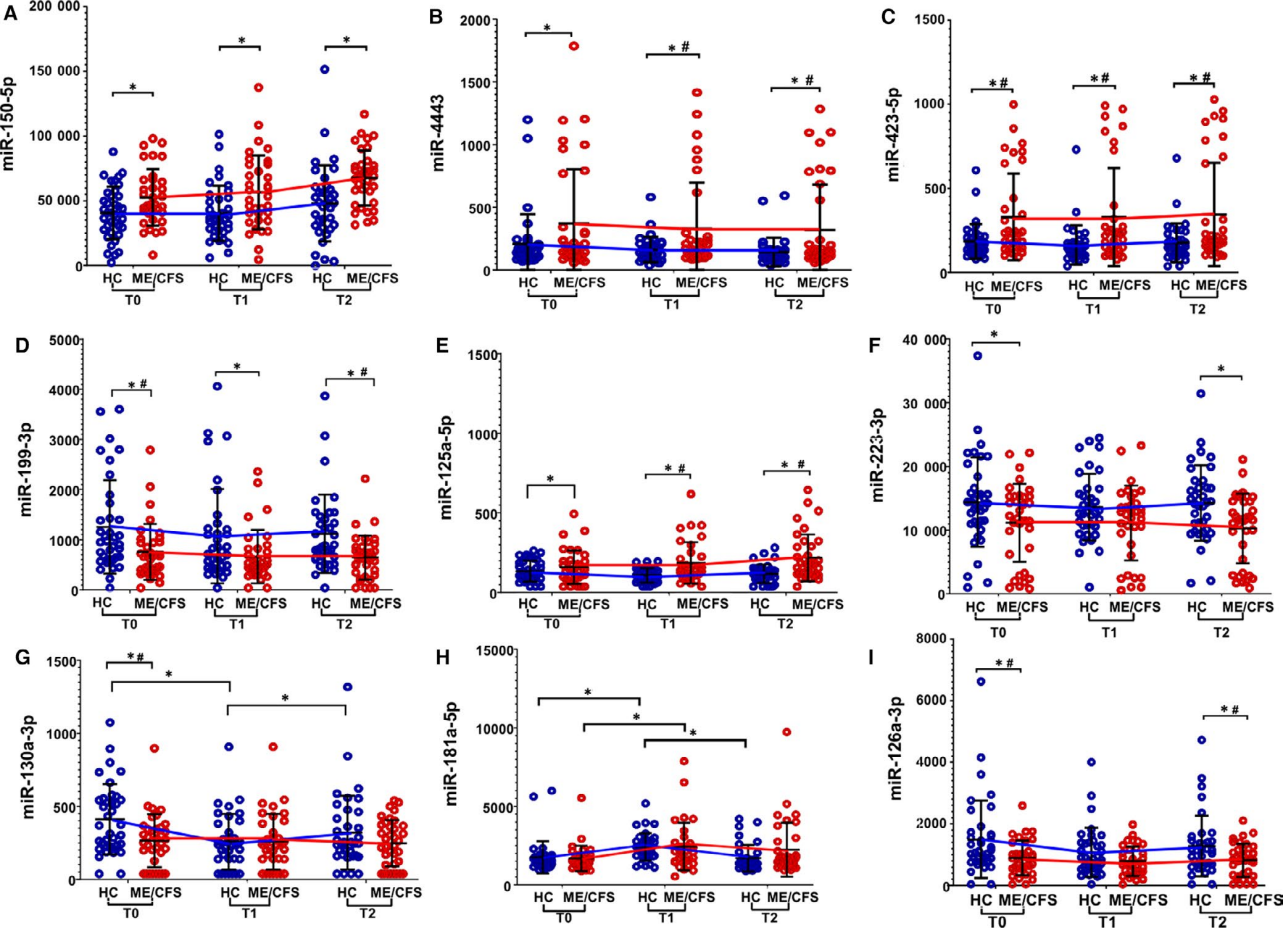
miRNA expression profile differences between diagnosed ME/CFS individuals and HCs in response to exercise challenge. Data are shown as normalized counts for ME/CFS (red) and healthy controls (HC; Blue) at T0, T1 and T2 (non‐fasting) for (A) miR‐150‐5p, (B) miR‐4443, (C) miR‐423‐5p, (D) miR‐199‐3p, (E) miR‐125a‐5p, (F) miR‐223‐3p (G) miR‐130a‐3p, (H) miR‐181a‐5p and (I) miR‐126a‐3p. * denotes *P *< .05 and # denotes FDR < 0.1

A subgroup of individuals who underwent exercise challenge was selected to compare differences in miRNA expression between ME/CFS and HCs in response to exercise (Figure 3), as detailed in 3.1. At T0, a total of 25 miRNAs were identified to be differentially expressed in ME/CFS compared to HCs with *P *< .05 (Table S2a), and six of them passed criteria of FDR < 0.1 significance. The expression of miR‐423‐5p was higher in individuals with ME/CFS compared with HCs, whereas miR‐22‐3p, miR‐199‐3p, miR‐126a‐3p, miR‐340‐5p, miR‐22‐3p and miR‐130a‐3p were underexpressed in ME/CFS compared with HCs (Figure 3). Among differentially expressed miRNAs with only *P < *.05, the expression of miR‐4443 and miR‐150‐5p was significantly elevated, whereas miR‐374‐5p, miR‐148a‐3p, miR‐146a‐5p and miR‐223‐3p expression was lower in ME/CFS than HC (Figure 3, Table S2a).

At T1 (VO_2_ max), 24 miRNAs were differentially expressed (*P < *.05) with seven passing FDR < 0.1 criteria between ME/CFS and HCs. Out of the miRNAs with FDR < 0.1, the expression of miR‐197‐3p, miR‐4443, miR‐423 (3p and 5p), miR‐1260a, miR‐125a‐5p, miR‐92a‐3p and miR‐331‐3p was higher in those with ME/CFS than HCs. The expression of miR‐4443 and miR‐22‐3p was reduced in individuals with ME/CFS than HC (Table S2b). Expression of miR‐150‐5p was higher, and miR‐199‐3p, miR‐146a‐5p and miR‐148‐3p were lower in ME/CFS than HCs but did not pass FDR < 0.1 criteria (*P* < .05).

A total of 33 miRNAs were differentially expressed (*P < .05*) in ME/CFS comparing to HC at T2 (recovery, four hours after T1) and twelve miRNAs passed criteria of FDR < 0.1 significance. Out of these twelve, miR‐197‐3p, miR‐4443, miR‐423 (3p and 5p), miR‐1260a, miR‐125a‐5p, miR‐92a‐3p and miR‐331‐3p showed higher expression in PBMCs of individuals with ME/CFS (Figure 3, Table S2c). Though FDR < 0.1 was not reached, miR‐150‐5p expression stayed higher in ME/CFS (*P *< .05). On the other hand, miR‐22‐3p, miR‐151a‐3p, miR‐199‐3p and miR‐126a‐3p showed decreased expression in ME/CFS comparing to HC at T2 (Table S2c). The expression of miR‐374b‐5p, miR‐148a‐3p and miR‐223‐3p also stayed lower at T2 in ME/CFS (*P *< .05), but FDR criteria was not reached.

Interesting to note is that regardless of time‐points being evaluated, individuals with ME/CFS showed higher expression of miR‐150‐5p and miR‐423, and reduced miR‐199‐3p levels, although not all miRNAs could reach FDR < 0.1 (Figure 3, Tables S2a‐c). The expression of some miRNAs was detected or fluctuated in response to exercise, namely (a) miR‐4443 expression fluctuated in response to exercise; higher at T0, reduced at T1 and back to elevated at T2, (b) miR‐125a‐5p which was overexpressed at T1, underexpressed at T2, but did not show significant differences at T0 and 3) miR‐374‐5p and miR‐148a‐5p were underexpressed both at T1 and T2 but were not significantly different at T0. It shows that expression of these miRNAs is affected by exercise in ME/CFS (Figure 3, Tables S2a‐c).

#### 3.4.2 Comparisons of miRNA expression between time‐points in exercise

The differential expression of miRNA in response to an exercise challenge was evaluated between ME/CFS and HCs for inter–time‐point comparisons (Figure 3). Ten miRNAs reached statistical significance of *P* < .05 for differential expression between T1 and T0 (T1/T0) in HCs (Table S3a), as compared to one in ME/CFS (Table S3b). No miRNA passed criteria of FDR < 0.1; however, miR‐181a‐5p showed increased expression at T1 regardless of disease status (Figure 3, Table S3a,b). Six miRNAs were differentially expressed between T2 and T1 (T2/T1) in HCs (*P < *.05); however, only miR‐363‐3p passed criteria of FDR < 0.1 with its expression being lower at T2 than at T1 (Table S3c). No miRNA passed criteria of FDR < 0.1 for T2/T1 comparison for ME/CFS, although miR‐4516 was significantly underexpressed at T2 (Table S3d). Interestingly, miR‐181a‐5p did not pass criteria of FDR < 0.1, the higher expression of miR‐181a‐5p seen in HCs and ME/CFS between T1 and T0 (*P* < .05), but lowered expression between T2 and T1, for HCs only (*P* < .05, Figure 3).

### 3.5 Response to exercise stratified by gender

#### 3.5.1 miRNA expression in response to exercise stratified by gender

**FIGURE 4:**
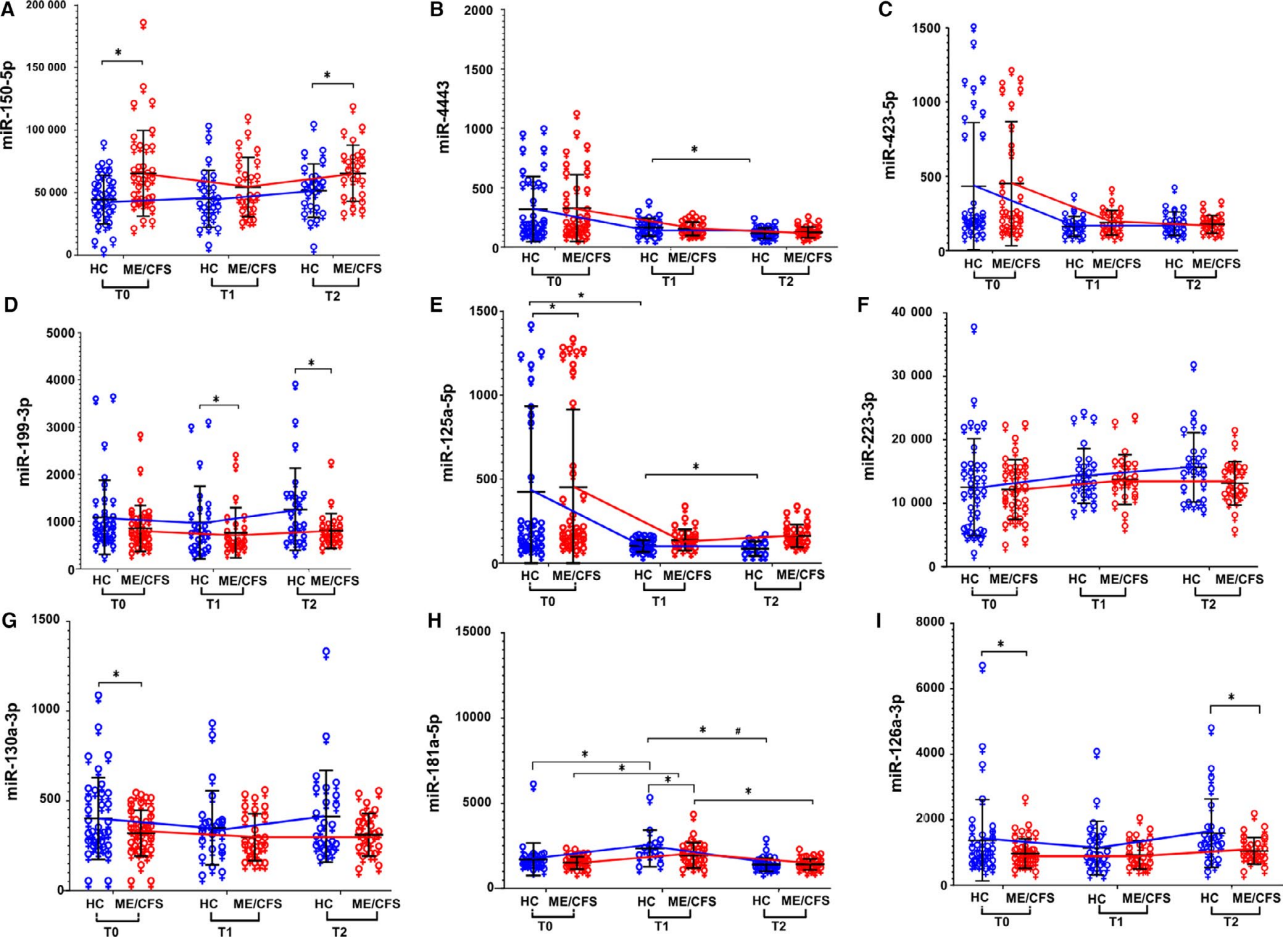
miRNA expression profile differences between diagnosed ME/CFS females and healthy females in response to exercise challenge Data are shown as normalized counts for ME/CFS (red ♀) and healthy females (HC; Blue ♀) at T0, T1 and T2 (non‐fasting) for (A) miR‐150‐5p, (B) miR‐4443, (C) miR‐423‐5p, (D) miR‐199‐3p, (E) miR‐125a‐5p, (F) miR‐223‐3p, (G) miR‐130a‐3p, (H) miR‐181a‐5p and (I) miR‐126a‐3p. * denotes *P* < .05 and # denotes FDR < 0.1

Females: None of the miRNAs passed criteria of FDR < 0.1 for analyses between ME/CFS females and HC females both at T1 and T2 (Figure 4). However, at T1, miR‐125a‐5p was overexpressed, whereas miR‐181a‐5p and miR‐146a‐5p were among those with reduced expression in ME/CFS females with *P < *.05 (Figure 4, Table S4a). At T2, miR‐146‐5p and miR‐150‐5p were among those with higher expression (*P < *.05), whereas miR‐199 (3p and 5p) and miR‐126a‐3p were among the miRNAs with reduced expression in ME/CFS females compared with HC females (*P *<* *.05; Table S4a,b).

**FIGURE 5:**
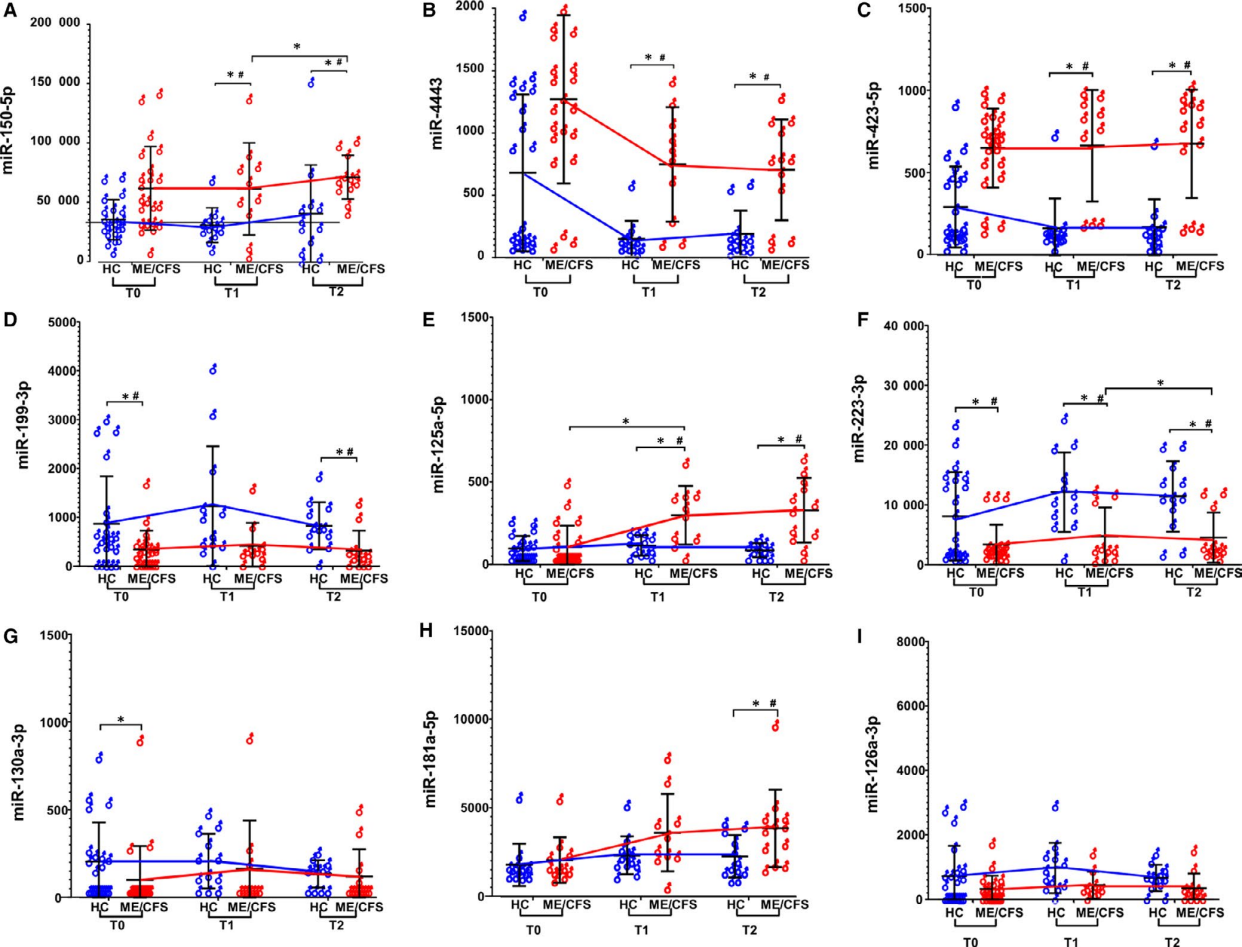
miRNA expression profile differences between diagnosed ME/CFS males and healthy males in response to exercise challenge Data are shown as normalized counts for ME/CFS (red ♂) and healthy males (HC; Blue ♂) at T0, T1 and T2 (non‐fasting) for (A) miR‐150‐5p, (B) miR‐4443, (C) miR‐423‐5p, (D) miR‐199‐3p, (E) miR‐125a‐5p, (F) miR‐223‐3p (G) miR‐130a‐3p, (H) miR‐181a‐5p and (I) miR‐126a‐3p. * denotes *P* < .05 and # denotes FDR < 0.1

Males: At T1, 36 miRNAs were differentially expressed between ME/CFS males and HC males and all passed criteria of FDR < 0.1. Selectively, the expression of miR‐423 (5p and 3p), miR‐4443, miR‐125a‐5p and miR‐150‐5p was higher in ME/CFS males, whereas miR‐374‐5p (a and b), miR‐22‐3p, miR‐223‐3p and miR‐146‐5p (a and b) were among those having reduced expression in ME/CFS males (Figure 5, Table S4c,). At T2, all 36 miRNAs that were differentially expressed between ME/CFS males and HC males passed criteria of FDR < 0.1. Out of the 36, miR‐423 (5p and 3p), miR‐125a‐5p, miR‐4443, miR‐150‐5p and miR‐181a‐5p were overexpressed, whereas miR‐22‐3p, miR‐374‐5p (a and b), miR‐199‐3p and miR‐223‐3p were reduced in ME/CFS males than HC males (Figure 5; Table S4d).

#### 3.5.2 Comparisons of miRNA expression between time‐points in exercise stratified by gender

The patterns of miRNA expression were further analysed in ME/CFS and HCs for differences between time‐points in both females and males. Eight miRNAs showed differential expression in healthy females for T1/T0, and only overexpressed miR‐363‐3p passed criteria of FDR < 0.1 (Table S5a). Both increased expression of miR‐181a‐5p and the reduced expression of miR‐125a‐5p could not reach FDR criteria (*P *< .05, Table S5a). For T2/T1 comparison in HC females, nine miRNAs were differentially expressed where miR‐125a‐5p was overexpressed and miR‐181a‐5p, miR‐363‐3p and miR‐4443 were reduced in expression. However, only miR‐181a‐5p, miR‐363‐3p passed criteria of FDR (<0.1) significance (*P* < .05, Table S5b).

In ME/CFS females, none passed criteria of FDR < 0.1 for T1/T0 comparison; however, miR‐181a‐5p showed increased expression (*P* < .05, Table S5c). For T2/T1 comparison, miR‐4516 expression was reduced (FDR < 0.1). Although the miR‐181a‐5p and miR‐363‐5p did not reach FDR < 0.1, they both showed decreased expression at T2 than T1 in ME/CFS females (*P* < .05), whereas both a‐ and b‐variants of miR‐146‐5p showed increased expression in ME/CFS females between T2 and T1 (*P* < .05, Table S4d).

For T1/T0 comparison for healthy males, none of the miRNAs passed criteria of FDR significance of 0.1. However, miR‐186‐5p expression increased between T1 and T0 but did not reach FDR < 0.1 (*P *< .05, Table S5e). No miRNA reached FDR criteria for T2/T1 comparison; expression of miR‐222‐3p expression was higher and miR‐16‐5p was lower in HC males (*P *< .05, Table S5f). ME/CFS males showed increased expression for miR‐125a‐5p and miR‐15b‐5p between T1 and T0, although it did not reach FDR < 0.1 (Table S5g). For T2/T1 comparison for ME/CFS males, 12 miRNAs were differentially expressed (*P* < .05) but only miR‐548q passed criteria of FDR < 0.1 significance with reduced expression (Table S5h). Although no other miRNA passed criteria of FDR < 0.1, increased expression of miR‐150‐5p and miR‐223‐3p was observed (Table S5h).

### 3.6 Fasting induced miRNA changes

The effects of fasting were assessed by evaluating differential expression of miRNAs separately in participants who ate unified breakfast after overnight fasting and those who did not eat breakfast after overnight fasting (at baseline only). When looking at comparison of miRNA expression between fasting ME/CFS and HCs, only six miRNAs showed differential expression with *P *<* *.05, out of which only three passed FDR criteria. The expression of miR‐150‐5p and miR‐342‐3p was higher, whereas miR‐548q expression was lower in ME/CFS than HCs (Table S6a). As discussed earlier, in participants who had unified breakfast, regardless of gender, the ME/CFS cohort had higher expression of miR‐423‐5p, miR‐4443 and miR‐150‐5p, whereas miR‐22‐3p, miR‐199‐3p,miR‐374b‐5p, miR‐126a‐3p and miR‐130a‐3p expression was reduced (Table S2a, Figure 3). Both miR‐342‐3p and miR‐548q were not among the 26 differentially expressed miRNAs between ME/CFS and HCs who had breakfast (non‐fasting) before blood draw. By contrast, miR‐150‐5p expression appears to be consistently higher in ME/CFS regardless of fasting status. No other differentially expressed miRNA overlapped. Moreover, the increased expression of miR‐150‐5p seen in ME/CFS regardless of fasting status, presented higher levels in fasting as compared to non‐fasting suggesting possible modulation in ME/CFS by dietary factors.

#### 3.6.1 miRNA expression in fasting and non‐fasting ME/CFS compared with HCs stratified by gender

Fasting ME/CFS females had higher miR‐150‐5p, miR‐342‐3p, miR‐222‐3p and miR‐223‐3p (*P* < .05) (Table S6b). However, only miR‐548q passed criteria of FDR < 0.1 with reduced expression. The expression of miR‐191‐5p, miR‐150‐5p, miR‐223‐3p and miR‐342‐3p also showed higher expression along with miR‐181a‐5p and miR‐423‐5p (*P *< .05) in fasting ME/CFS males although only few passed FDR criteria < 0.1 (Table S6c). MiR‐548q was consistently lower in fasting ME/CFS males which also passed the FDR criteria (Table S6c).

The non‐fasting ME/CFS females showed six miRNAs as differentially expressed from healthy females (*P *< .05), with none reaching FDR < 0.1. The expression of miR‐150‐5p was higher, whereas expression of miR‐126a‐3p, miR‐130a‐3p and miR‐374b‐5p was lower in ME/CFS than healthy females (Figure 4, Table S6d). In non‐fasting ME/CFS males, 48 miRNAs were differentially expressed from healthy males and all were underexpressed (*P *< .05). All of them passed criteria of FDR < 0.1, namely miR‐374‐5p (a and b), miR‐223‐3p, miR‐199a‐3p, miR‐146‐5p (a and b), miR‐148a‐3p, miR‐222‐3p and miR‐191‐5p (Figure 5, Table S6e).

Three miRNAs were highlighted here that showed opposite pattern of expression in ME/CFS between fasting and non‐fasting: (a) the fasting ME/CFS females and males showed overexpressed miR‐223‐3p opposite to consistently underexpressed pattern in non‐fasting ME/CFS, even in response to exercise, (b) miR‐191‐5p was overexpressed in both fasting females and males but it was underexpressed in non‐fasting ME/CFS males, (c) the consistent higher expression of miR‐343‐3p in fasting ME/CFS which was not identified in other group and (d) underexpressed miR‐548q in both fasting ME/CFS females and males.

## 4 DISCUSSION

This is the first ME/CFS exercise challenge study which evaluated gender differences in ME/CFS utilizing miRNA expression profiles. The results of this study have highlighted: (a) miRNAs that are differentially expressed in ME/CFS, (b) altered miRNAs induced by exercise challenge in ME/CFS, (c) gender influenced miRNAs and their differential expression in response to exercise in disease state and finally (d) fasting induced miRNA difference, emphasizing the possible role they may play in the ME/CFS‐associated disease biology.

In this study, miR‐150‐5p, miR‐130a‐3p, miR‐199‐3p, miR‐223‐3p, miR‐126a‐3p, miR‐4443, miR‐374‐5p, miR‐146a‐5p and miR‐423 have emerged as prominent miRNAs whose expression was altered in ME/CFS. These miRNAs with genes involved in cellular processes and immunity as their molecular targets have been detected in other ME/CFS studies.^10^, ^11^, ^12^, ^13^, ^14^, ^15^, ^16^, ^17^, ^18^, ^19^, ^20^, ^27^ Brenu et al had detected miR‐146a‐5p, miR‐223‐3p and miR‐126a‐3p in NK and CD8 + cells, and in plasma of individuals with ME/CFS, respectively.^15^, ^16^ Bjersing et al detected reduced expression of both miR‐223‐3p and miR‐374‐5p in cerebrospinal fluid and serum of ME/CFS subjects.^17^, ^27^

The up‐regulation of miR‐150‐5p is seen in both T‐cell and B‐cell maturation and differentiation via Notch pathway and c‐Myb, respectively, and it influences the release of pro‐inflammatory cytokines suggesting its critical involvement in the modulation of immunity and inflammatory response.^28^, ^29^, ^30^

MiR‐199‐3p, regulated by free fatty acids and adipokines, is a negative regulator of NF‐κB and IL‐8.^31^ Additionally, low miR‐199‐3p expression, seen in ME/CFS subjects, is linked with poor survival outcome in carcinomas possibly affecting the disease‐related physiological burden.^32^, ^33^

Another dysregulated miR‐223 identified in this study is reported in other inflammatory conditions, infections and carcinomas.^34^ It modulates the TLR4/TLR2/NF‐κB/STAT3 signalling pathway consequently affecting inflammatory cytokine expression^35^ and controls inflammatory bowel disease (IBD)‐associated intestinal inflammation by inhibiting NLRP3 inflammasome.^36^

It was shown that the cytokines released in response to inflammatory assault, particularly TNF‐α, is directly suppressed by miR‐130a‐3p, reducing inflammation and associated oxidative stress.^37^ The higher inflammatory cytokine expression seen in ME/CFS could therefore be induced by diminished miR‐130a‐3p expression in ME/CFS subjects, as identified in the above‐mentioned study.^37^

Another miRNA presenting consistent reduced expression both at baseline and at recovery post‐exercise is miR‐126a‐3p, which is linked with inflammation. However, Petty et al^19^ reported that individuals with ME/CFS had up‐regulated miR‐126. The cause of this conflicting finding could be attributed to the subgroup differences within the ME/CFS subject population, possible gender influences due to higher proportion of females in Petty’ study or lifestyle habits. On the other hand, we observed elevated expression of miR‐423 in ME/CFS which is associated with inflammation and is also reported to be overexpressed by Petty et al.^19^ In a study by Gharbi et al,^38^ miR‐423‐3p was found to be elevated in veterans exposed to sulphur mustard (SM), a vesicant agent responsible for SM‐induced necrosis and inflammation.

Of the highlighted miRNAs in this study, miR‐146a is among the first‐studied miRNA for its role as key regulator of the immune and inflammatory response. Diminished miR‐146a expression leads to overexpression of STAT1 and reduced IFN‐γ secretion, resulting in loss of repressive effect of regulatory T lymphocytes (Treg).^39^

miR‐374a‐5p regulates the expression of ubiquitin ligase, mTOR signalling pathway and monocyte chemoattractant protein (MCP)‐1, critical in inflammatory and immune response.^40^, ^41^

The overexpressed miR‐4443 increases pro‐inflammatory cytokines by activated NF‐κΒ pathway via targeting TRAF4.^41^ In fact, a consistent feature of ME/CFS is significantly reduced natural killer (NK) cell function, unique pro‐ and anti‐inflammatory cytokines, hypersensitivity responses and viral infections prior to ME/CFS‐associated symptom onset.

In relation to exercise response, we successfully identified key miRNAs which could be critical to the post‐exertional recovery‐associated pathways in ME/CFS. Consistently in ME/CFS, miR‐150‐5p and miR‐423 (3p and 5p) were overexpressed while miR‐199a‐3p, miR‐130a‐3p, miR‐146a‐3p and miR‐223‐3p were underexpressed regardless of stage in the exercise challenge. Additionally, the expression of miR‐4443 was transiently reduced in ME/CFS after exercise.

Most of the miRNAs which were found to be reduced in ME/CFS, regardless of gender and time‐point otherwise confer protective effect against inflammation.^42^, ^43^, ^44^ Inter‐time‐point comparisons highlighted miR‐181a‐5p, miR‐125a‐5p and miR‐363‐3p to be influenced by gender. These miRNAs are associated with TNF‐α levels, regulation of differential activation of macrophages and inflammation and mast cells function, respectively, whose expression is affected by exercise.^44^, ^45^, ^46^, ^47^ In this sense, the observed miRNA expression pattern in ME/CFS could be interpreted as an “echo” of the imbalance in immune molecules reported in former ME/CFS biomarker studies.^1^, ^2^, ^3^, ^4^

The reduction of miR‐22‐3p has been reported in ME/CFS at recovery post‐exercise by Baraniuk et al, who evaluated the exercise induced changes in cerebrospinal fluid miRNAs in Gulf War Illness and ME/CFS.^20^ We saw this reduction only in males, and interestingly, the group that displaced this reduction in response to exercise in Baraniuk study was mostly males.^20^ MiR‐22 directly targets NLRP3 (Nod‐like receptor protein 3), a key protein in the NLRP3 inflammasome.^48^

The HCs recovered from exercise induced down‐regulation of both miR‐130‐3p and miR‐223 miRNAs in response to exercise challenge, but it was also only reflected in males.

Few studies have evaluated the relationship of fasting with differential expression of miRNAs, but the risk diet imposes on chronic disease development is well known. In fasting ME/CFS, we identified key miRNAs that may play a critical regulatory role in ME/CFS metabolic state. The inconsistencies in miRNA expression profiles between studies could have been, at least partly, a result of fasting‐induced processes influencing miRNA expression. It is, therefore, extremely important to align assessment and reporting with Common Data Elements (CDE) in human subject research that improves data quality and allows for comparisons across multiple studies. NIH has recently adopted CDE principles in detailed reporting of ME/CFS subjects’ conditions and encourages the use of these carefully selected CDEs in ME/CFS research which should be utilized for proper registry towards generalizability of findings.^49^

To the best of our knowledge, we are the first to characterize the gender effects on miRNA profiles in ME/CFS compared with matched healthy controls, particularly in response to exercise. The findings of this study should be useful not only for the understanding of gender‐dependent disease‐specific ME/CFS pathology, but also in the identification of therapeutic regimes suited to ME/CFS gender‐specific biology demands.

## CONFLICT OF INTEREST

The authors confirm that there is no conflict of interest.

## AUTHOR CONTRIBUTION

LN conceived and designed the experiments; FC, EO, EAP, EMM, JA, JCM, MAF and NK provided clinical data; LS performed the experiments; MB, AC and LN contributed to data analysis; AC, LN, EO, JCM, JA, NK and MF wrote and reviewed the manuscript.

## ETHICAL APPROVAL AND CONSENT TO PARTICIPATE

The research was carried out according to the World Medical Association Declaration of Helsinki. All subjects signed an informed consent approved by the appropriate human subject protections committees (Institutional Review Board of the Nova Southeastern University (NSU) and Miami Veterans Affairs Healthcare system Research Development Committee for Florida, USA; Public Health Research Ethics Committee DGSP‐CSISP for Valencia, Spain, and Clinical Ethics Committee of the Vall d'Hebron University Hospital for Barcelona, Spain).

## Supporting information

Appendix S1

## Data Availability

The data that support the findings of this study are available in the Appendix S1 of this article.

## References

[1] Fletcher MA , Zeng XR , Maher K , et al. Biomarkers in chronic fatigue syndrome: evaluation of natural killer cell function and dipeptidyl peptidase IV/CD26. PLoS ONE. 2010;5:e10817. 20520837 10.1371/journal.pone.0010817 PMC2876037

[2] Fletcher MA , Zeng XR , Barnes Z , et al. Plasma cytokines in women with chronic fatigue syndrome. J Transl Med. 2009;7:96. 19909538 10.1186/1479-5876-7-96 PMC2779802

[3] Klimas NG , Broderick G , Fletcher MA . Biomarkers for chronic fatigue. Brain Behav Immun. 2012;26:1202‐1210. 22732129 10.1016/j.bbi.2012.06.006 PMC5373648

[4] Broderick G , Fuite J , Kreitz A , et al. A formal analysis of cytokine networks in chronic fatigue syndrome. Brain Behav Immun. 2010;24:1209‐1217. 20447453 10.1016/j.bbi.2010.04.012 PMC2939140

[5] Craddock TJ , Fritsch P , Rice MA Jr , et al. A role for homeostatic drive in the perpetuation of complex chronic illness: Gulf War Illness and chronic fatigue syndrome. PLoS ONE. 2014;9:e84839. 24416298 10.1371/journal.pone.0084839 PMC3885655

[6] Carruthers BM , Jain AK , De Meirleir KL , et al. Myalgic encephalomyelitis/chronic fatigue syndrome: clinical working case definition, diagnostic and treatment protocols. J Chronic Fatigue Syndrome. 2003;11:7‐115.

[7] Perez M , Jaundoo R , Hilton K , et al. Genetic predisposition for pmmune system, hormone, and metabolic dysfunction in Myalgic Encephalomyelitis/Chronic Fatigue Syndrome: a pilot study. Front Pediatr. 2019;7:206. 31179255 10.3389/fped.2019.00206 PMC6542994

[8] Almenar‐Pérez E , Ovejero T , Sánchez‐Fito T , et al. Epigenetic components of myalgic encephalomyelitis/chronic fatigue syndrome uncover potential transposable element activation. Clin Ther. 2019;41(4):675‐698. 30910331 10.1016/j.clinthera.2019.02.012

[9] Jeffrey MG , Nathanson L , Aenlle K , et al. Treatment avenues in myalgic encephalomyelitis/chronic fatigue syndrome: a split‐gender pharmacogenomic study of gene‐expression modules. Clin Ther. 2019;41:815‐835.e6. 30851951 10.1016/j.clinthera.2019.01.011

[10] Brenu EW , van Driel ML , Staines DR , et al. Immunological abnormalities as potential biomarkers in chronic fatigue syndrome/myalgic encephalomyelitis. J Transl Med. 2011;9:81. 21619669 10.1186/1479-5876-9-81 PMC3120691

[11] Momen‐Heravi F , Bala S . miRNA regulation of innate immunity. J Leukoc Biol. 2018;103:1205‐1217. 10.1002/JLB.3MIR1117-459R 29656417

[12] Nejad C , Stunden HJ , Gantier MP . A guide to miRNAs in inflammation and innate immune responses. FEBS J. 2018;285:3695‐3716. 29688631 10.1111/febs.14482

[13] Mehta A , Baltimore D . MicroRNAs as regulatory elements in immune system logic. Nat Rev Immunol. 2016;16:279. 27121651 10.1038/nri.2016.40

[14] Almenar‐Pérez E , Sánchez‐Fito T , Ovejero T , et al. Impact of polypharmacy on candidate biomarker mirnomes for the diagnosis of fibromyalgia and myalgic encephalomyelitis/chronic fatigue syndrome: striking back on treatments. Pharmaceutics. 2019;11:126. 10.3390/pharmaceutics11030126 PMC6471415 30889846

[15] Brenu EW , Ashton KJ , van Driel M , et al. Cytotoxic lymphocyte microRNAs as prospective biomarkers for chronic fatigue syndrome/myalgic encephalomyelitis. J Affect Disord. 2012;141:261‐269. 22572093 10.1016/j.jad.2012.03.037

[16] Brenu EW , Ashton KJ , Batovska J , et al. High‐throughput sequencing of plasma microRNA in chronic fatigue syndrome/myalgic encephalomyelitis. PLoS ONE. 2014;9:e102783. 25238588 10.1371/journal.pone.0102783 PMC4169517

[17] Bjersing JL , Lundborg C , Bokarewa MI , Mannerkorpi K . Profile of cerebrospinal microRNAs in fibromyalgia. PLoS ONE. 2013;8:e78762. 24205312 10.1371/journal.pone.0078762 PMC3808359

[18] Cerdá‐Olmedo G , Mena‐Durán AV , Monsalve V , Oltra E . Identification of a microRNA signature for the diagnosis of fibromyalgia. PLoS ONE. 2015;10:e0121903. 25803872 10.1371/journal.pone.0121903 PMC4372601

[19] Petty RD , McCarthy NE , Le Dieu R , Kerr JR . MicroRNAs hsa‐miR‐99b, hsa‐miR‐330, hsa‐miR‐126 and hsa‐miR‐30c: potential diagnostic biomarkers in natural killer (NK) cells of patients with chronic fatigue syndrome (CFS)/myalgic encephalomyelitis (ME). PLoS ONE. 2016;11:e0150904. 26967895 10.1371/journal.pone.0150904 PMC4788442

[20] Baraniuk JN , Shivapurkar N . Exercise–induced changes in cerebrospinal fluid miRNAs in Gulf War Illness, Chronic Fatigue Syndrome and sedentary control subjects. Sci Rep. 2017;7:15338. 29127316 10.1038/s41598-017-15383-9 PMC5681566

[21] Wallis A , Butt H , Ball M , et al. Support for the microgenderome invites enquiry into gender differences. Gut Microbes. 2017;8:46‐52. 27808584 10.1080/19490976.2016.1256524 PMC5361606

[22] Smylie AL , Broderick G , Fernandes H , et al. A comparison of gender‐specific immune signatures in Gulf War Illness and chronic fatigue syndrome. BMC Immunol. 2013;14:29. 23800166 10.1186/1471-2172-14-29 PMC3698072

[23] Fukuda K , Straus SE , Hickie I , et al. The chronic fatigue syndrome: a comprehensive approach to its definition and study. Ann Intern Med. 1994;121:953‐959. 7978722 10.7326/0003-4819-121-12-199412150-00009

[24] Reeves WC , Jones JF , Maloney E , et al. Prevalence of chronic fatigue syndrome in metropolitan, urban, and rural Georgia. Populat Health Metrics. 2007;5:1. 10.1186/1478-7954-5-5 PMC1904178 17559660

[25] Semler G , Witichen H , Joschke K , et al. Test‐retest reliability of a standardized psychiatric interview (DIS/CIDI). Eur Arch Psychiatry Neurol Sci. 1987;236:214‐222. 3582430 10.1007/BF00383851

[26] Storey JD , Tibshirani R . Statistical significance for genomewide studies. Proc Natl Acad Sci USA. 2003;100:9440‐9445. 12883005 10.1073/pnas.1530509100 PMC170937

[27] Bjersing JL , Bokarewa MI , Mannerkorpi K . Profile of circulating microRNAs in fibromyalgia and their relation to symptom severity: an exploratory study. Rheumatol Int. 2015;35:635‐642. 25261961 10.1007/s00296-014-3139-3

[28] Kroesen B , Teteloshvili N , Smigielska‐Czepiel K , et al. Immuno‐miRs: critical regulators of T‐cell development, function and ageing. Immunology. 2015;144:1‐10. 25093579 10.1111/imm.12367 PMC4264905

[29] Zhou B , Wang S , Mayr C , et al. miR‐150, a microRNA expressed in mature B and T cells, blocks early B cell development when expressed prematurely. Proc Natl Acad Sci USA. 2007;104:7080‐7085. 17438277 10.1073/pnas.0702409104 PMC1855395

[30] Ying W , Tseng A , Chang RC , et al. miR‐150 regulates obesity‐associated insulin resistance by controlling B cell functions. Sci Rep. 2016;6:20176. 26833392 10.1038/srep20176 PMC4735333

[31] Gu N , You L , Shi C , et al. Expression of miR‐199a‐3p in human adipocytes is regulated by free fatty acids and adipokines. Mol Med Rep. 2016;14:1180‐1186. 27279151 10.3892/mmr.2016.5379 PMC4940088

[32] Nam EJ , Yoon H , Kim SW , et al. MicroRNA expression profiles in serous ovarian carcinoma. Clin Cancer Res. 2008;14:2690‐2695. 18451233 10.1158/1078-0432.CCR-07-1731

[33] Ecke TH , Stier K , Weickmann S , et al. miR‐199a‐3p and miR‐214‐3p improve the overall survival prediction of muscle‐invasive bladder cancer patients after radical cystectomy. Cancer Med. 2017;6:2252‐2262. 28879675 10.1002/cam4.1161 PMC5633587

[34] Haneklaus M , Gerlic M , O'Neill LA , Masters S . miR‐223: infection, inflammation and cancer. J Intern Med. 2013;274:215‐226. 23772809 10.1111/joim.12099 PMC7166861

[35] Wu J , Niu P , Zhao Y , et al. Impact of miR‐223‐3p and miR‐2909 on inflammatory factors IL‐6, IL‐1ß, and TNF‐α, and the TLR4/TLR2/NF‐κB/STAT3 signaling pathway induced by lipopolysaccharide in human adipose stem cells. PLoS ONE. 2019;14:e0212063. 30807577 10.1371/journal.pone.0212063 PMC6391004

[36] Neudecker V , Haneklaus M , Jensen O , et al. Myeloid‐derived miR‐223 regulates intestinal inflammation via repression of the NLRP3 inflammasome. J Exp Med. 2017;214:1737‐1752. 28487310 10.1084/jem.20160462 PMC5460990

[37] Jiang Y , Wang W , Liu Z , et al. Overexpression of miR‐130a‐3p/301a‐3p attenuates high glucose‐induced MPC5 podocyte dysfunction through suppression of TNF‐α signaling. Exp Therapeut Med. 2018;15:1021‐1028. 10.3892/etm.2017.5465 PMC5772991 29434693

[38] Gharbi S , Shamsara M , Khateri S , et al. Identification of reliable reference genes for quantification of MicroRNAs in serum samples of sulfur mustard‐exposed Veterans. Cell J. 2015;17:494‐501. 26464821 10.22074/cellj.2015.9 PMC4601870

[39] Williams AE , Perry MM , Moschos SA , et al. Role of miRNA‐146a in the regulation of the innate immune response and cancer. Biochem Soc Trans. 2008;36(6):1211‐1215. 19021527 10.1042/BST0361211

[40] Unterbruner K , Matthes F , Schilling J , et al. MicroRNAs miR‐19, miR‐340, miR‐374 and miR‐542 regulate MID1 protein expression. PLoS ONE. 2018;13:e0190437. 29293623 10.1371/journal.pone.0190437 PMC5749791

[41] Yang Z , Guo Z , Dong J , et al. miR‐374a regulates inflammatory response in diabetic nephropathy by targeting MCP‐1 expression. Front Pharmacol. 2018;9:900. 30147653 10.3389/fphar.2018.00900 PMC6095963

[42] Qi Y , Zhou Y , Chen X , et al. Microrna‐4443 causes cD4 T cells dysfunction by targeting TnFr‐associated Factor 4 in graves’ disease. Front Immunol. 2017;8:1440. 29163513 10.3389/fimmu.2017.01440 PMC5671953

[43] Bhatt K , Lanting LL , Jia Y , et al. Anti‐inflammatory role of MicroRNA‐146a in the pathogenesis of diabetic nephropathy. J Am Soc Nephrol. 2016;27:2277‐2288. 26647423 10.1681/ASN.2015010111 PMC4978034

[44] Wang J , Bai X , Song Q , et al. miR‐223 inhibits lipid deposition and inflammation by suppressing toll‐like receptor 4 signaling in macrophages. Int J Mol Sci. 2015;16:24965‐24982. 26492242 10.3390/ijms161024965 PMC4632784

[45] Marques‐Rocha JL , Samblas M , Milagro FI , et al. Noncoding RNAs, cytokines, and inflammation‐related diseases. FASEB J. 2015;29:3595‐3611. 26065857 10.1096/fj.14-260323

[46] Xie W , Li Z , Li M , et al. miR‐181a and inflammation: miRNA homeostasis response to inflammatory stimuli in vivo. Biochem Biophys Res Commun. 2013;430:647‐652. 23220232 10.1016/j.bbrc.2012.11.097

[47] Radom‐Aizik S , Zaldivar F Jr , Leu S , et al. Effects of exercise on microRNA expression in young males peripheral blood mononuclear cells. Clin Transl Sci. 2012;5:32‐38. 22376254 10.1111/j.1752-8062.2011.00384.x PMC4664183

[48] Chen Y , Wang L , Pitzer AL , et al. Contribution of redox‐dependent activation of endothelial Nlrp3 inflammasomes to hyperglycemia‐induced endothelial dysfunction. J Mol Med. 2016;94:1335‐1347. 27783111 10.1007/s00109-016-1481-5 PMC5512566

[49] National Institute of Health . Common Data Element (CDE) Resource Portal. 2019 https://www.nlm.nih.gov/cde/index.html

