# Supplementary material for: Unravelling myalgic encephalomyelitis/chronic fatigue syndrome (ME/CFS): Gender‐specific changes in the microRNA expression profiling in ME/CFS
Source: J Cell Mol Med. 2020 Apr 14;24(10):5865–77. doi: 10.1111/jcmm.15260 (PMC7214164; doi:10.1111/jcmm.15260)
Supplement: Supplementary file 1 — Appendix S1 [file JCMM-24-5865-s001.doc]

**Supplementary information:**

**Measures**

*Short-Form 36-item Health Survey (SF-36):* In this study, the SF-36 questionnaire was used to compare individuals with ME/CFS and matched HCs for eight domains well-being: physical functioning, physical role functioning, bodily pain, general health perception, vitality, social functioning, emotional role and mental health. Spanish version of SF-36 was used for Spanish speaking participants [1].

*Graded exercise testing protocol:* After uniform breakfast (yogurt and banana), the subjects rested in a sitting position (easy chair) for 30 minutes prior to the initial blood draw (T0). Immediately after the blood draw, exercise challenge was performed. The protocol used for the selected subjects was a standard maximal Graded eXercise Test (GXT) according to McArdle´s protocol [2]. Participating subjects pedaled at an initial output of 60W for 2 min, followed by an increase of 30W every 2 min until the subject reached: (1) a plateau in maximal oxygen consumption (VO2); (2) a respiratory exchange ratio >1.15; or (3) the subject stopped the test. Second and third blood draws were conducted upon reaching peak effort (VO2 max) (T1) and at 4 hours post-exercise (T2), respectively.

References

1. **Alonso J, Prieto L, Antó J.** La versión española del SF-36 Health Survey (Cuestionario de Salud SF-36): un instrumento para la medida de los resultados clínicos. Med Clin. 1995; 104: 771-6.
2. **McArdle WD, Katch FI, Katch VL.***Exercise physiology: energy, nutrition, and human performance.*1991.

**Table S1a: miRNAs differentially expressed between ME/CFS and HCs irrespective of sex and fasting status at T0**

| **miRNA ID** | ***P* value** | **Fold-Change** |
| --- | --- | --- |
| ***Over expressed*** | | |
| hsa-miR-150-5p* | < .001 | 1.56 |
| hsa-miR-4443 | .033 | 1.51 |
| hsa-miR-423-5p | .036 | 1.39 |
| hsa-miR-342-3p | .040 | 1.23 |
| ***Under expressed*** | | |
| hsa-miR-199-3p | .009 | -1.53 |
| hsa-miR-126a-3p | .020 | -1.56 |
| hsa-let-7i-5p | .017 | -1.41 |
| hsa-miR-130a-3p | .026 | -1.39 |

**denotes the miRNA that reached FDR significance.*

**Table S1b: miRNAs differentially expressed between ME/CFS and healthy females irrespective of and fasting status at T0**

| **miRNA ID** | ***P* value** | **Fold-Change** |
| --- | --- | --- |
| ***Over expressed*** | | |
| hsa-miR-150-5p | .003 | 1.47 |
| hsa-miR-342-3p | .009 | 1.33 |

**denotes the miRNA that reached FDR significance.*

**Table S1c: miRNAs differentially expressed between ME/CFS and healthy males irrespective of fasting status at T0**

| **miRNA ID** | ***P* value** | **Fold-Change** |
| --- | --- | --- |
| ***Over expressed*** | | |
| hsa-miR-423-5p* | < .001 | 2.23 |
| hsa-miR-296-5p* | .004 | 2.54 |
| hsa-miR-4443* | .005 | 1.86 |
| hsa-miR-150-5p | .003 | 1.74 |
| ***Under expressed*** | | |
| hsa-miR-223-3p* | .009 | -2.39 |
| hsa-miR-199-3p* | .022 | -2.50 |
| hsa-miR-16-5p* | .022 | -2.05 |
| hsa-miR-142-3p* | .023 | -2.16 |
| hsa-let-7g-5p* | .028 | -1.97 |
| hsa-miR-26a-5p | .021 | -1.63 |
| hsa-miR-23a-3p | .035 | -1.61 |

**denotes the miRNA that reached FDR significance.*

**Table S2a: miRNAs differentially expressed between ME/CFS and HCs who ate breakfast, irrespective of sex (T0).**

| **miRNA ID** | ***P* value** | **Fold-Change** |
| --- | --- | --- |
| ***Over expressed*** |  |  |
| hsa-miR-423-5p* | .002 | 1.79 |
| hsa-miR-4443 | .045 | 1.79 |
| hsa-miR-150-5p | .018 | 1.29 |
| ***Under expressed*** |  |  |
| hsa-miR-22-3p* | .009 | -1.76 |
| hsa-miR-199-3p* | .007 | -1.66 |
| hsa-miR-374b-5p | .008 | -1.43 |
| hsa-miR-126a-3p* | .011 | -1.66 |
| hsa-miR-340-5p* | .011 | -1.66 |
| hsa-miR-130a-3p* | .005 | -1.54 |
| hsa-miR-374a-5p | .009 | -1.45 |
| hsa-miR-148a-3p | .003 | -1.44 |
| hsa-miR-146a-5p | .009 | -1.42 |
| hsa-miR-19a-3p | .007 | -1.42 |
| hsa-let-7i-5p | .002 | -1.38 |
| hsa-miR-106b-5p | .004 | -1.37 |
| hsa-miR-28-5p | .003 | -1.33 |
| hsa-miR-20-5p | .006 | -1.32 |
| hsa-miR-23a-3p | .005 | -1.29 |
| hsa-miR-223-3p | .041 | -1.29 |
| hsa-miR-18a-5p | .005 | -1.28 |
| hsa-miR-19b-3p | .042 | -1.28 |
| hsa-miR-98-5p | .029 | -1.25 |
| hsa-miR-24-3p | .029 | -1.25 |
| hsa-miR-301a-3p | .026 | -1.21 |

**denotes the miRNA that reached FDR significance*.

**Table S2b: miRNA differentially expressed between ME/CFS and HCs at peak of exercise challenge (VO_2_ max, T1) irrespective of sex**

| **miRNA ID** | ***P* value** | **Fold-Change** |
| --- | --- | --- |
| ***Over expressed*** | | |
| hsa-miR-421* | .008 | 2.06 |
| hsa-miR-423-5p* | .010 | 2.03 |
| hsa-miR-423-3p* | .002 | 2.00 |
| hsa-miR-125a-5p* | .001 | 1.73 |
| hsa-miR-891a-5p* | .011 | 1.57 |
| hsa-miR-150-5p | .008 | 1.39 |
| hsa-miR-125b-5p | .020 | 1.32 |
| hsa-miR-15b-5p | .025 | 1.23 |
| ***Under expressed*** | | |
| hsa-miR-4443* | .017 | -1.81 |
| hsa-miR-22-3p* | < .001 | -1.71 |
| hsa-miR-199-3p | .031 | -1.60 |
| hsa-miR-19a-3p | .010 | -1.41 |
| hsa-miR-28-5p | < .001 | -1.38 |
| hsa-let-7f-5p | .004 | -1.36 |
| hsa-miR-374a-5p | .001 | -1.35 |
| hsa-miR-148a-3p | .015 | -1.34 |
| hsa-miR-146a-5p | .009 | -1.33 |
| hsa-let-7i-5p | .001 | -1.30 |
| hsa-miR-26a-5p | .005 | -1.29 |
| hsa-miR-23a-3p | .013 | -1.26 |
| hsa-miR-93-5p | .012 | -1.24 |
| hsa-let-7e-5p | .022 | -1.24 |
| hsa-miR-185-5p | .026 | -1.23 |
| hsa-miR-4536-5p | .028 | -1.15 |

**denotes the miRNA that reached FDR significance.*

**Table S2c: miRNA differentially expressed between ME/CFS and HCs at recovery post exercise challenge (T2) irrespective of sex**

| **miRNA ID** | ***P* value** | **Fold-Change** |
| --- | --- | --- |
| ***Over expressed*** | | |
| hsa-miR-197-3p* | .037 | 2.41 |
| hsa-miR-4443* | .006 | 2.24 |
| hsa-miR-423-3p* | .006 | 2.09 |
| hsa-miR-1260a* | .009 | 2.08 |
| hsa-miR-423-5p* | .003 | 1.95 |
| hsa-miR-125a-5p* | < .001 | 1.84 |
| hsa-miR-92a-3p* | .028 | 1.58 |
| hsa-miR-331-3p* | .018 | 1.53 |
| hsa-miR-361-3p | .003 | 1.46 |
| hsa-miR-15b-5p | .002 | 1.42 |
| hsa-miR-150-5p | .001 | 1.41 |
| hsa-miR-342-3p | .035 | 1.23 |
| ***Under expressed*** | | |
| hsa-miR-22-3p* | .005 | -2.30 |
| hsa-miR-151a-3p* | .019 | -2.01 |
| hsa-miR-199-3p* | .002 | -1.73 |
| hsa-miR-126a-3p* | .015 | -1.56 |
| hsa-miR-26a-5p | .002 | -1.49 |
| hsa-miR-374b-5p | .001 | -1.44 |
| hsa-miR-185-5p | < .001 | -1.44 |
| hsa-miR-23a-3p | .001 | -1.39 |
| hsa-miR-148a-3p | .007 | -1.39 |
| hsa-miR-340-5p | .027 | -1.39 |
| hsa-miR-223-3p | .004 | -1.38 |
| hsa-miR-19a-3p | .019 | -1.37 |
| hsa-miR-28-5p | .009 | -1.35 |
| hsa-miR-148b-3p | .024 | -1.34 |
| hsa-miR-23b-3p | .003 | -1.33 |
| hsa-let-7i-5p | .003 | -1.30 |
| hsa-let-7f-5p | .044 | -1.28 |
| hsa-miR-24-3p | .044 | -1.25 |
| hsa-let-7e-5p | .010 | -1.23 |
| hsa-miR-361-5p | .030 | -1.22 |
| hsa-miR-454-3p | .016 | -1.19 |

**denotes the miRNA that reached FDR significance.*

**Table S3a: Comparison between peak of exercise challenge (VO_2_ max) and baseline (T1 vs T0) for miRNA expression in HCs.**

| **miRNA ID** | ***P* value** | **Fold-Change** |
| --- | --- | --- |
| ***Over expressed*** | | |
| hsa-miR-363-3p | < .001 | 1.43 |
| hsa-miR-181a-5p | .004 | 1.37 |
| hsa-miR-26b-5p | .033 | 1.22 |
| hsa-miR-342-3p | .038 | 1.22 |
| hsa-miR-186-5p | .026 | 1.25 |
| hsa-miR-28-5p | .033 | 1.18 |
| hsa-miR-454-3p | .006 | 1.16 |
| ***Under expressed*** | | |
| hsa-miR-130a-3p | .039 | -1.36 |
| hsa-miR-125a-5p | .045 | -1.25 |
| hsa-let-7g-5p | .045 | 1.16 |

**denotes the miRNA that reached FDR significance.*

**Table S3b: Comparison between peak of exercise challenge (VO_2_ max) and baseline (T1 vs T0) for miRNA expression in ME/CFS.**

| **miRNA ID** | ***P* value** | **Fold-Change** |
| --- | --- | --- |
| ***Over expressed*** | | |
| hsa-miR-181a-5p | .012 | 1.44 |

**denotes the miRNA that reached FDR significance.*

**Table S3c: Comparison between recovery (4 hours post peak) and peak of exercise challenge (VO_2_ max) (T2 vs T1) for miRNA expression in HCs.**

| **miRNA ID** | ***P* value** | **Fold-Change** |
| --- | --- | --- |
| ***Over expressed*** | | |
| hsa-miR-26a-5p | .040 | 1.25 |
| ***Under expressed*** | | |
| hsa-miR-363-3p* | < .001 | -1.50 |
| hsa-miR-181a-5p | < .001 | -1.41 |
| hsa-miR-16-5p | .023 | -1.29 |
| hsa-miR-29a-3p | .013 | -1.28 |
| hsa-miR-25-3p | .022 | -1.23 |

**denotes the miRNA that reached FDR significance.*

**Table S3d: Comparison between recovery (4 hours post peak) and peak of exercise challenge (VO_2_ max) (T2 vs T1) for miRNA expression in ME/CFS.**

| **miRNA ID** | ***P* value** | **Fold-Change** |
| --- | --- | --- |
| ***Over expressed*** | | |
| hsa-miR-4516 | .032 | -1.54 |

**denotes the miRNA that reached FDR significance.*

**Table S4a: miRNA differentially expressed between ME/CFS and healthy females at peak of exercise challenge (VO_2_ max, T1)**

| **miRNA ID** | ***P* value** | **Fold-Change** |
| --- | --- | --- |
| ***Over expressed*** | | |
| hsa-miR-125a-5p | .020 | 1.34 |
| ***Under expressed*** | | |
| hsa-miR-181a-5p | .027 | -1.25 |
| hsa-miR-146a-5p | .044 | -1.22 |
| hsa-miR-28-5p | .005 | -1.21 |
| hsa-miR-361-5p | .017 | -1.14 |

**denotes the miRNA that reached FDR significance*

**Table S4b:** **miRNA differentially expressed between ME/CFS and healthy females at recovery (T2)**

| **miRNA ID** | ***P* value** | **Fold-Change** |
| --- | --- | --- |
| ***Over expressed*** | | |
| hsa-miR-146-5p | < .001 | 1.42 |
| hsa-miR-29a-3p | .044 | 1.29 |
| hsa-miR-342-3p | .006 | 1.28 |
| hsa-miR-150-5p | .032 | 1.27 |
| hsa-miR-25-3p | .018 | 1.19 |
| ***Under expressed*** | | |
| hsa-miR-199-3p | .022 | -1.56 |
| hsa-miR-126a-3p | .023 | -1.51 |
| hsa-miR-199-5p | .010 | -1.48 |
| hsa-miR-23b-3p | < .001 | -1.38 |
| hsa-miR-185-5p | .003 | -1.35 |
| hsa-miR-221-3p | .038 | -1.30 |
| hsa-miR-148b-3p | .047 | -1.34 |

**denotes the miRNA that reached FDR significance*

**Table S4c:** **miRNA differentially expressed between ME/CFS and healthy males at peak of exercise challenge (VO2 max, T1)**

| **miRNA ID** | ***P* value** | **Fold-Change** |
| --- | --- | --- |
| ***Over expressed*** | | |
| hsa-miR-4443* | < .001 | 4.90 |
| hsa-miR-423-3p* | .002 | 4.08 |
| hsa-miR-423-5p* | < .001 | 4.07 |
| hsa-miR-4536-5p* | .038 | 3.50 |
| hsa-miR-197-3p* | .028 | 3.25 |
| hsa-miR-125a-5p* | .003 | 2.58 |
| hsa-miR-92a-3p* | .029 | 2.19 |
| hsa-miR-150-5p* | .019 | 1.98 |
| hsa-miR-361-3p* | .039 | 1.79 |
| hsa-miR-15b-5p* | .0047 | 1.68 |
| ***Under expressed*** | | |
| hsa-miR-19a-3p* | < .001 | -4.42 |
| hsa-miR-29b-3p* | .004 | -4.16 |
| hsa-miR-19b-3p* | < .001 | -3.83 |
| hsa-miR-374a-5p* | .003 | -3.54 |
| hsa-let-7f-5p* | .001 | -3.36 |
| hsa-miR-146b-5p* | .007 | -3.20 |
| hsa-miR-22-3p* | < .001 | -3.14 |
| hsa-miR-21-5p* | .002 | -2.93 |
| hsa-miR-223-3p* | .008 | -2.53 |
| hsa-miR-98-5p* | .002 | -2.46 |
| hsa-miR-26b-5p* | .003 | -2.39 |
| hsa-miR-148a-3p* | .002 | -2.28 |
| hsa-miR-374b-5p* | .003 | -2.27 |
| hsa-miR-29c-3p* | .023 | -2.20 |
| hsa-miR-30b-5p* | .019 | -2.17 |
| hsa-let-7g-5p* | .009 | -2.16 |
| hsa-miR-106b-5p* | .002 | -2.12 |
| hsa-let-7i-5p* | .002 | -2.11 |
| hsa-miR-20-5p* | .014 | -2.11 |
| hsa-miR-15a-5p* | .021 | -2.08 |
| hsa-miR-28-5p* | .013 | -2.02 |
| hsa-miR-26a-5p* | < .001 | -1.94 |
| hsa-miR-146a-5p* | .030 | -1.97 |
| hsa-let-7e-5p* | .007 | -1.74 |
| hsa-miR-454-3p* | < .001 | -1.71 |
| hsa-miR-23a-3p* | .033 | -1.71 |

**denotes the miRNA that reached FDR significance*

**Table S4d:** **miRNA differentially expressed between ME/CFS and healthy males at recovery (T2)**

| **miRNA ID** | ***P value*** | **Fold-Change** |
| --- | --- | --- |
| ***Over expressed*** | | |
| hsa-miR-423-5p* | < .001 | 4.00 |
| hsa-miR-423-3p* | < .001 | 3.79 |
| hsa-miR-125a-5p* | < .001 | 3.78 |
| hsa-miR-4443* | < .001 | 3.64 |
| hsa-miR-197-3p* | .006 | 3.58 |
| hsa-miR-1260a* | .001 | 3.01 |
| hsa-miR-92a-3p* | .004 | 2.67 |
| hsa-miR-15b-5p* | < .001 | 2.12 |
| hsa-miR-361-3p* | .007 | 2.04 |
| hsa-miR-150-5p* | .026 | 1.75 |
| hsa-miR-181a-5p* | .038 | 1.68 |
| ***Under expressed*** | | |
| hsa-miR-22-3p* | .020 | -6.11 |
| hsa-miR-29b-3p* | .003 | -4.83 |
| hsa-miR-21-5p* | < .001 | -3.64 |
| hsa-miR-19a-3p* | .004 | -2.94 |
| hsa-miR-26b-5p* | .002 | -2.87 |
| hsa-miR-146b-5p* | .015 | -2.83 |
| hsa-miR-19b-3p* | .009 | -2.69 |
| hsa-miR-374a-5p* | .008 | -2.64 |
| hsa-miR-199-3p* | .010 | -2.54 |
| hsa-miR-374b-5p* | .004 | -2.51 |
| hsa-miR-223-3p* | .003 | -2.50 |
| hsa-let-7f-5p* | .033 | -2.46 |
| hsa-miR-26a-5p* | .008 | -2.39 |
| hsa-let-7i-5p* | < .001 | -2.37 |
| hsa-let-7g-5p* | .009 | -2.36 |
| hsa-miR-28-5p* | .011 | -2.35 |
| hsa-miR-146a-5p* | .011 | -2.22 |
| hsa-miR-15a-5p* | .041 | -2.12 |
| hsa-miR-98-5p* | .021 | -2.04 |
| hsa-miR-148a-3p* | .012 | -2.04 |
| hsa-miR-23a-3p* | .002 | -1.88 |
| hsa-miR-186-5p* | .022 | -1.83 |
| hsa-miR-185-5p* | .040 | -1.74 |
| hsa-miR-222-3p* | .011 | -1.65 |

**denotes the miRNA that reached FDR significance*

**Table S5a: Comparison between peak of exercise challenge (VO2 max) and baseline (T1 vs T0) for miRNA expression in healthy females.**

| **miRNA ID** | ***P* value** | **Fold-Change** |
| --- | --- | --- |
| ***Over expressed*** | | |
| hsa-miR-363-3p* | < .001 | 1.45 |
| hsa-miR-181a-5p | .005 | 1.42 |
| hsa-miR-140-5p | .047 | 1.37 |
| hsa-miR-29a-3p | .025 | 1.28 |
| hsa-let-7g-5p | .042 | 1.18 |
| hsa-miR-454-3p | .011 | 1.17 |
| ***Under expressed*** | | |
| hsa-miR-125a-5p | .036 | -1.30 |
| hsa-miR-221-3p | .036 | -1.25 |

**denotes the miRNA that reached FDR significance.*

**Table S5b: Comparison between 4 hours post peak of exercise challenge (VO2 max) and peak of exercise (T2 vs T1) for miRNA expression in healthy females.**

| **miRNA ID** | ***P value*** | **Fold-Change** |
| --- | --- | --- |
| ***Over expressed*** | | |
| hsa-miR-125a-5p | .034 | 1.30 |
| hsa-miR-26a-5p | .046 | 1.27 |
| ***Under expressed*** | | |
| hsa-miR-181a-5p* | < .001 | -1.73 |
| hsa-miR-363-3p* | < .001 | -1.56 |
| hsa-miR-4516 | .002 | -1.48 |
| hsa-miR-1260a | .005 | -1.47 |
| hsa-miR-4443 | .007 | -1.42 |
| hsa-miR-361-3p | .013 | -1.27 |
| hsa-miR-29a-3p | .039 | -1.26 |

**denotes the miRNA that reached FDR significance.*

T**able S5c: Comparison between peak of exercise challenge (VO2 max) and baseline (T1 vs T0) for miRNA expression in ME/CFS females.**

| **miRNA ID** | ***P value*** | **Fold-Change** |
| --- | --- | --- |
| ***Over expressed*** | | |
| hsa-miR-181a-5p | .014 | 1.28 |
| ***Under expressed*** | | |
| hsa-let-7e-5p | .007 | -1.32 |

**denotes the miRNA that reached FDR significance.*

**Table S5d: Comparison between 4 hours post peak of exercise challenge (VO2 max) and peak of exercise (T2 vs T1) for miRNA expression in ME/CFS females.**

| **miRNA ID** | ***P value*** | **Fold-Change** |
| --- | --- | --- |
| ***Over expressed*** | | |
| hsa-miR-146a-5p | .001 | 1.24 |
| hsa-miR-146b-5p | .031 | 1.20 |
| ***Under expressed*** | | |
| hsa-miR-4516* | .001 | -1.58 |
| hsa-miR-181a-5p | .002 | -1.37 |
| hsa-miR-363-3p | .020 | -1.27 |

**denotes the miRNA that reached FDR significance.*

**Table S5e: Comparison between peak of exercise challenge (VO2 max) and baseline (T1 vs T0) for miRNA expression in healthy male controls.**

| **miRNA ID** | ***P value*** | **Fold-Change** |
| --- | --- | --- |
| ***Over expressed*** | | |
| hsa-miR-186-5p | .0012 | 1.49 |

**denotes the miRNA that reached FDR significance.*

**Table S5f: Comparison between 4 hours post peak of exercise challenge (VO2 max) and peak of exercise (T2 vs T1) for miRNA expression in healthy male controls.**

| **miRNA ID** | ***P value*** | **Fold-Change** |
| --- | --- | --- |
| ***Over expressed*** | | |
| hsa-miR-222-3p | .042 | 1.44 |
| ***Under expressed*** | | |
| hsa-miR-16-5p | .048 | -1.67 |

**denotes the miRNA that reached FDR significance*.

**Table S5g: Comparison between peak of exercise challenge (VO2 max) and baseline (T1 vs T0) for miRNA expression in ME/CFS males.**

| **miRNA ID** | ***P value*** | **Fold-Change** |
| --- | --- | --- |
| ***Over expressed*** | | |
| hsa-miR-125a-5p | .047 | 2.00 |
| hsa-miR-15b-5p | .050 | 1.49 |

**denotes the miRNA that reached FDR significance.*

**Table S5h: Comparison between 4 hours post peak of exercise challenge (VO2 max) and peak of exercise (T2 vs T1) for miRNA expression in ME/CFS males.**

| **miRNA ID** | ***P value*** | **Fold-Change** |
| --- | --- | --- |
| ***Over expressed*** | | |
| hsa-miR-222-3p | .015 | 2.12 |
| hsa-miR-150-5p | .022 | 1.91 |
| hsa-miR-342-3p | .021 | 1.90 |
| hsa-let-7g-5p | .020 | 1.54 |
| hsa-miR-223-3p | .011 | 1.48 |
| hsa-miR-191-5p | .031 | 1.41 |
| hsa-miR-23a-3p | .037 | 1.33 |
| hsa-miR-93-5p | .042 | 1.29 |
| ***Under expressed*** | | |
| hsa-miR-451a | .027 | -1.95 |
| hsa-miR-549a | .047 | -1.89 |
| hsa-miR-548q* | .001 | -1.82 |
| hsa-miR-1290 | .044 | -1.71 |

**denotes the miRNA that reached FDR significance*

**Table S6a: miRNA differentially expressed between fasting ME/CFS and HCs at baseline.**

| **miRNA ID** | ***P value*** | **Fold-Change** |
| --- | --- | --- |
| ***Over expressed*** | | |
| hsa-miR-150-5p* | < .001 | 1.96 |
| hsa-miR-342-3p* | .007 | 1.73 |
| hsa-let-7g-5p | .020 | 1.54 |
| hsa-miR-142-3p | .021 | 1.24 |
| ***Under expressed*** | | |
| hsa-miR-548q* | .006 | -1.89 |
| hsa-miR-644a | .041 | -1.74 |

**denotes the miRNA that reached FDR significance*

**Table S6b: miRNA differentially expressed between fasting ME/CFS and healthy females at baseline.**

| **miRNA ID** | ***P value*** | **Fold-Change** |
| --- | --- | --- |
| ***Over expressed*** | | |
| hsa-miR-222-3p | .015 | 2.12 |
| hsa-miR-150-5p | .022 | 1.91 |
| hsa-miR-342-3p | .021 | 1.90 |
| hsa-miR-223-3p | .011 | 1.48 |
| hsa-miR-191-5p | .031 | 1.41 |
| hsa-miR-23a-3p | .037 | 1.33 |
| hsa-miR-93-5p | .042 | 1.29 |
| ***Under expressed*** | | |
| hsa-miR-451a | .027 | -1.95 |
| hsa-miR-548q* | .001 | -1.82 |
| hsa-miR-1290 | .044 | -1.71 |
| hsa-miR-590-5p | .049 | -1.50 |

**denotes the miRNA that reached FDR significance*.

**Table S6c: miRNA differentially expressed between fasting ME/CFS and healthy males at baseline.**

| **miRNA ID** | ***P value*** | **Fold-Change** |
| --- | --- | --- |
| ***Over expressed*** | | |
| hsa-miR-296-5p* | .019 | 2.83 |
| hsa-miR-191-5p* | .002 | 2.15 |
| hsa-miR-150-5p* | .009 | 2.06 |
| hsa-let-7g-5p | .052 | 1.75 |
| hsa-miR-181a-5p | .022 | 1.70 |
| hsa-miR-15b-5p | .031 | 1.67 |
| hsa-miR-423-5p | .028 | 1.58 |
| hsa-miR-342-3p | .029 | 1.57 |
| hsa-miR-223-3p* | .001 | 1.56 |
| hsa-miR-23a-3p | < .001 | 1.44 |
| hsa-miR-142-3p | .036 | 1.41 |
| hsa-let-7b-5p | .041 | 1.37 |
| ***Under expressed*** | | |
| hsa-miR-378h* | < .001 | -3.06 |
| hsa-miR-548q* | .013 | -2.06 |
| hsa-miR-30e-5p | .001 | -1.47 |
| hsa-miR-888-5p | .036 | -1.43 |

**denotes the miRNA that reached FDR significance*.

**Table S6d: miRNA differentially expressed between non-fasting ME/CFS and healthy females at baseline.**

| **miRNA ID** | ***P value*** | **Fold-Change** |
| --- | --- | --- |
| ***Over expressed*** | | |
| hsa-miR-92a-3p | .038 | 1.35 |
| hsa-miR-150-5p | .048 | 1.27 |
| hsa-miR-454-3p | .012 | 1.16 |
| ***Under expressed*** | | |
| hsa-miR-126a-3p | .045 | -1.56 |
| hsa-miR-130a-3p | .005 | -1.48 |
| hsa-miR-374b-5p | .047 | -1.19 |

**denotes the miRNA that reached FDR significance.*

**Table S6e: miRNA differentially expressed between non-fasting ME/CFS and healthy control males at baseline.**

| **miRNA ID** | ***P value*** | **Fold-Change** |
| --- | --- | --- |
| ***Under expressed*** | | |
| hsa-miR-374a-5p* | .001 | -3.82 |
| hsa-miR-21-5p* | .001 | -3.77 |
| hsa-miR-19b-3p* | < .001 | -3.45 |
| hsa-miR-19a-3p* | < .001 | -3.32 |
| hsa-miR-29b-3p* | .009 | -3.13 |
| hsa-miR-22-3p* | .005 | -2.97 |
| hsa-miR-20-5p* | .002 | -2.79 |
| hsa-miR-146b-5p* | .007 | -2.76 |
| hsa-let-7f-5p* | .003 | -2.75 |
| hsa-miR-223-3p* | .003 | -2.71 |
| hsa-miR-340-5p* | .007 | -2.66 |
| hsa-miR-374b-5p* | .002 | -2.61 |
| hsa-miR-29c-3p* | .006 | -2.58 |
| hsa-miR-98-5p* | .002 | -2.55 |
| hsa-miR-199-3p* | .037 | -2.50 |
| hsa-miR-146a-5p* | .009 | -2.47 |
| hsa-let-7i-5p* | .004 | -2.42 |
| hsa-miR-28-5p* | .001 | -2.41 |
| hsa-miR-106b-5p* | .001 | -2.41 |
| hsa-miR-148a-3p* | .002 | -2.38 |
| hsa-miR-15a-5p* | .004 | -2.37 |
| hsa-miR-32-5p* | .004 | -2.28 |
| hsa-miR-29a-3p* | .011 | -2.25 |
| hsa-miR-483-3p* | .013 | -2.23 |
| hsa-miR-142-3p* | .014 | -2.20 |
| hsa-let-7g-5p* | .010 | -2.11 |
| hsa-let-7a-5p* | .022 | -2.05 |
| hsa-miR-16-5p* | .024 | -2.01 |
| hsa-miR-30b-5p* | .018 | -1.88 |
| hsa-miR-23a-3p* | .010 | -1.84 |
| hsa-miR-140-5p* | .013 | -1.82 |
| hsa-miR-26a-5p* | .011 | -1.82 |
| hsa-miR-222-3p* | .013 | -1.79 |
| hsa-miR-24-3p* | .041 | -1.76 |
| hsa-miR-301a-3p* | .001 | -1.74 |
| hsa-miR-26b-5p* | .035 | -1.67 |
| hsa-let-7e-5p* | .005 | -1.59 |
| hsa-miR-424-5p* | .001 | -1.56 |
| hsa-miR-454-3p* | < .001 | -1.53 |
| hsa-miR-18a-5p* | .011 | -1.52 |
| hsa-miR-191-5p* | .047 | -1.51 |
| hsa-miR-23b-3p* | .027 | -1.50 |

*** *denotes the miRNA that reached FDR significance.*
